# Travel Time to Methadone Treatment Via Personal Vehicle vs Public Transit

**DOI:** 10.1001/jamanetworkopen.2025.57361

**Published:** 2026-02-03

**Authors:** Benjamin A. Howell, Junghwan Kim, Thomas A. Thornhill, Jinhyung Lee, Emma T. Biegacki, Lauretta E. Grau, David A. Fiellin, Robert Heimer, Gregg S. Gonsalves

**Affiliations:** 1 Section of General Internal Medicine, Yale School of Medicine, New Haven, Connecticut; 2 Program in Addiction Medicine, Yale School of Medicine, New Haven, Connecticut; 3 Department of Geography, College of Natural Resources and Environment, Virginia Tech, Blacksburg; 4 Department of Epidemiology of Microbial Diseases, Yale School of Public Health, New Haven, Connecticut; 5 Public Health Modeling Unit, Yale School of Public Health, New Haven, Connecticut; 6 Department of Geography and Environment, Faculty of Social Science, Western University, London, Ontario, Canada; 7 Department of Health Policy and Management, Yale School of Public Health, New Haven, Connecticut

## Abstract

**Importance:**

The requirement for in-person, often daily, attendance at opioid treatment programs (OTPs) makes travel times a barrier to methadone treatment. Research on methadone accessibility has primarily focused on travel via personal vehicle, and there is uncertainty about public transit travel time to methadone treatment.

**Objective:**

To estimate travel time via personal vehicle vs public transit to methadone treatment in the state of Connecticut.

**Design, Setting, and Participants:**

This cross-sectional study included geospatial analysis of median travel time to nearest OTP via personal vehicle and public transit from all census block groups (CBGs). This study took place in the state of Connecticut in 2023. Participants were all CBGs in Connecticut.

**Exposures:**

Participants were characterized by racial and ethnic demographics; household income; car ownership; urban, suburban, or rural designations; and per-capita opioid overdose deaths.

**Main Outcomes and Measures:**

The primary outcome was the median travel time to nearest OTP by via personal vehicle and public transit. Spatial error models using *k*-nearest neighbor spatial weight matrices were estimated to assess the associations between sociodemographic characteristics and travel times for each transportation mode (personal vehicle vs public transit) at the CBG level.

**Results:**

From the centroids of the 2702 CBGs in Connecticut, the median (IQR) travel time to the closest OTP was 11.0 (7.5-16.3) minutes by personal vehicle and 41.7 (31.0-49.5) minutes via public transit, with 1431 CBGs (53%) lacking access to public transit or having high public transit times (>60 minutes or no trip available). Travel times via public transit increased along the urban-rural gradient and across CBGs with an increasing percentage of non-Hispanic White residents. Median (IQR) travel times to an OTP from the 489 CBGs with the highest per-capita overdose death rates were 8.2 (5.9-11.7) minutes by personal vehicle and 37.6 (27.8-48.5) minutes by public transit, with 166 (34%) lacking public transit access.

**Conclusions and Relevance:**

The findings of this cross-sectional study of barriers to access to methadone treatment suggest that areas with high overdose death rates, low car ownership, and high public transit travel times should be targets for interventions (eg, mobile services or greater use of take-home doses for patients) to lower travel-based barriers to methadone. Current federal statutes and regulations governing methadone provision are the greatest barrier, as they directly require often daily transit to opioid treatment clinics. Reducing this barrier requires policy changes.

## Key Points

**Question:**

How does transportation access to methadone treatment for opioid use disorder via personal vehicle vs public transit vary across different demographic groups?

**Findings:**

This cross-sectional study estimated travel time for 2702 census block groups in Connecticut to the nearest opioid treatment program via personal vehicle vs public transit. Median travel times from the census block groups with the highest per-capita overdose death rates were 8.2 minutes by personal vehicle and 37.6 minutes by public transit, with 34% lacking adequate public transit access.

**Meaning:**

These findings suggest that transportation access to methadone treatment via public transit is poor and that areas with high overdose death rates, low car ownership, and high public transit travel times should be targets for interventions to lower travel-based barriers to methadone treatment.

## Teaser

This cross-sectional study investigates the travel time via personal vehicle vs public transit to methadone treatment alongside census block demographics in Connecticut.

## Introduction

The US is experiencing an opioid overdose crisis, with over 80 000 opioid-involved overdose deaths in 2023.^1^ Medications for opioid use disorder (MOUD) substantially reduce overdose deaths, yet only approximately 25% of people with an opioid use disorder (OUD) received MOUDs in 2022.^2,3,4,5,6^ Three MOUDs are currently approved by the US Food and Drug Administration: buprenorphine, methadone, and naltrexone. Methadone has the longest history of clinical use and strong evidence supporting its use across multiple outcomes, including reduced nonprescribed opioid use, improved treatment retention, decreased overdose risk, and superior clinical results among fentanyl users.^7,8,9^ Yet, methadone treatment is the most regulated of these medications, both by federal and state agencies, with access largely limited to brick-and-mortar Substance Abuse and Mental Health Services Administration–certified opioid treatment programs (OTPs).^10,11^ Given methadone’s superior clinical evidence and its regulated status, there have been increasing calls to ensure access to high-quality methadone treatment.^12,13^

Methadone treatment, particularly in the first few weeks, requires in-person evaluation for initiation of treatment and daily in-person medication dispensing at OTP facilities that often have limited service hours.^9^ The combination of limited locations and daily in-person treatment protocols creates access barriers, and poor transportation access can worsen those barriers.^8^ Transportation time to methadone increases the indirect costs of treatment and decreases the likelihood of treatment initiation and retention.^14,15,16,17^ Understanding and mapping transportation barriers to OTPs is crucial to better address challenges to methadone access for people with OUD. Such information is needed to optimize locations for future OTPs, to assess the impact of extending hours of existing facilities, and to understand the role of novel delivery methods (eg, office-based methadone and mobile methadone units).

To date, most studies on transportation access to OTPs have focused on analyses of drive times using a personal vehicle.^14,18,19,20,21,22,23^ Geospatial analyses of transportation access by personal vehicle do not account for the barriers faced by people who rely on public transit. While most people use personal vehicles for daily transportation in the US, almost 9% of households report no vehicle ownership.^24^ The lack of vehicle ownership and reliance on public transit, specifically buses, is also concentrated in households with lower household incomes.^24,25^ People with OUD often have fewer financial resources, relative to people without OUD, and a greater likelihood of relying on public transit for daily transportation needs. The few studies that have looked at public transit access to methadone treatment were from outside the US,^26^ where methadone provision is regulated differently, or analyzed public transit access at the city or county level but not the state level.^27,28^ Furthermore, studies on transportation access to OTPs have used aggregated populations for larger geographic areas, such as counties or zip codes, which can obscure heterogeneity in travel times across smaller areas within these jurisdictions. Finally, there are evaluations of drive vs public transit time to buprenorphine clinics (or OTPs), but work in this area has been limited to a few urban centers.^29^

This study seeks to address these gaps in the research on travel burdens for people with OUD, by using census block groups (CBGs) in the state of Connecticut as points of origin and comparing access to OTPs by both personal vehicle and public transit. Connecticut has a high burden of opioid overdose deaths and a range of urban and rural geographies, along with representative demographic and financial distribution, including low-income communities. Using demographic characteristics and geocoded overdose death incidence, we also identify areas of the state that have high rates of opioid overdose deaths, low rates of car ownership, and poor public transit–based access to opioid treatment programs.

## Methods

In this cross-sectional study, we conducted a geospatial analysis of OTP access by personal vehicle and public transit in Connecticut. Because this analysis used publicly available data and no individual level data, it was not considered human participants research and did not require institutional review board review or informed consent, in accordance with 45 CFR §46. This study follows the Strengthening the Reporting of Observational Studies in Epidemiology (STROBE) reporting guideline for cohort studies.

We first obtained from the Connecticut Department of Mental Health and Addiction Services the addresses for all 29 outpatient facilities approved to dispense methadone for the treatment of OUD in Connecticut at the time of our analysis. We included all CBGs in Connecticut. CBGs are the smallest geographic area (600-3000 residents living in each CBG) where detailed sociodemographic information was available from the US Census Bureau’s American Community Survey (ACS) 2022 5-Year Data. For sociodemographic data, we included estimates of the percentage of households with incomes below the federal poverty level and percentage of Hispanic, non-Hispanic Black (hereafter, Black), and non-Hispanic White (hereafter, White) residents. As the ACS transportation-related variable—percentage of households with 1 or more cars—is not available at the CBG level, we used census tract–level data as the most appropriate alternative. We used the National Center for Education Statistics Locale Classification data to categorize CBGs across the urban-suburban-rural gradient. Finally, we used data from the Connecticut Office of Chief Medical Examiner (OCME) on the residential address of all accidental and undetermined opioid-involved overdose decedents from 2019 to 2021 to calculate the CBG level per-capita opioid-involved overdose death rate. We excluded any decedents who did not have a residential address in the OCME dataset. Data were provided by the OCME through an ongoing collaboration between the Yale School of Public Health and the OCME on overdose mortality. Per-capita opioid-involved overdose death rate was used as a proxy for OUD treatment need at the CBG level.

### Estimation of Personal Vehicle and Public Transit Travel Times

Our primary outcome of interest was an estimate of the minimum travel time from centroid of a CBG to the nearest OTP. To estimate minimum travel time and to account for the variation in travel time within a CBG, we divided Connecticut into 3000 feet by 3000 feet grid cells—a granular spatial unit where we can reasonably assume travel time is homogeneous—and calculated the minimum travel time to an OTP. For each grid cell–OTP pair, we calculated travel times via both personal vehicle and public transit using a departure time of 8 am on a Wednesday. This departure time was used to approximate travel on a typical weekday during operating hours of an OTP. For personal vehicle travel time, we employed the Google Maps API in October to November 2023, a widely used tool that leverages real-world street networks and historical traffic data to generate driving time estimates.^30^ For public transit travel times, using static General Transit Feed Specification datasets, we built a high-resolution, schedule-aware transit network for Connecticut. We have described the generation and use of this public transit dataset elsewhere.^31^ Travel times calculated at the grid cell level were aggregated to the CBG level by averaging the travel times of all grid cells contained within each block group.

### Statistical Analysis

We generated descriptive data of median (IQR) travel times via personal vehicle and via public transit for all CBGs and by the previously described CBG characteristic described. We also reported the percentage of all CBGs, overall and by CBG characteristics, that did not have a public transit option to an OTP available. Spatial error models using *k*-nearest neighbor (*k* = 5) spatial weight matrices were estimated to assess the associations between sociodemographic characteristics and travel times for each transportation mode (model 1, personal vehicle; and model 2, public transit) at the CBG level (eMethods in Supplement 1). For the public transit model, CBGs without access to transit services—defined as having travel times exceeding 60 minutes or no available transit trips—were assigned to a value equal to 1.5 times the maximum observed travel time to represent no access conditions. This approach was used in lieu of excluding these observations, as over half of the CBGs fell into the no access category.

For data visualization, we generated isochrone maps representing travel time to at least 1 OTP from CBGs via personal vehicle or public transit, respectively. In these maps, CBGs are colored to represent groupings at which travel to at least 1 OTP is within 10-minute intervals. In the map for public transit, we also marked CBGs where no public transit trip to an OTP was available or excessive lengthy trips (>60 minutes). We generated cumulative incidence plots across personal vehicle and public transit travel times weighted by urban-rural CBG classification and by racial and ethnic subgroups, plotting the cumulative proportion of individuals with a specific travel time or shorter by their respective classifications. All geospatial analyses were conducted in ArcGIS Pro version 3.1.3 (Esri), and statistical analyses were conducted in R statistical software version 4.3.0 (R Project for Statistical Computing). Statistical significance was assessed using 2-sided tests with a significance threshold of *P* < .05.

## Results

**Figure 1.**
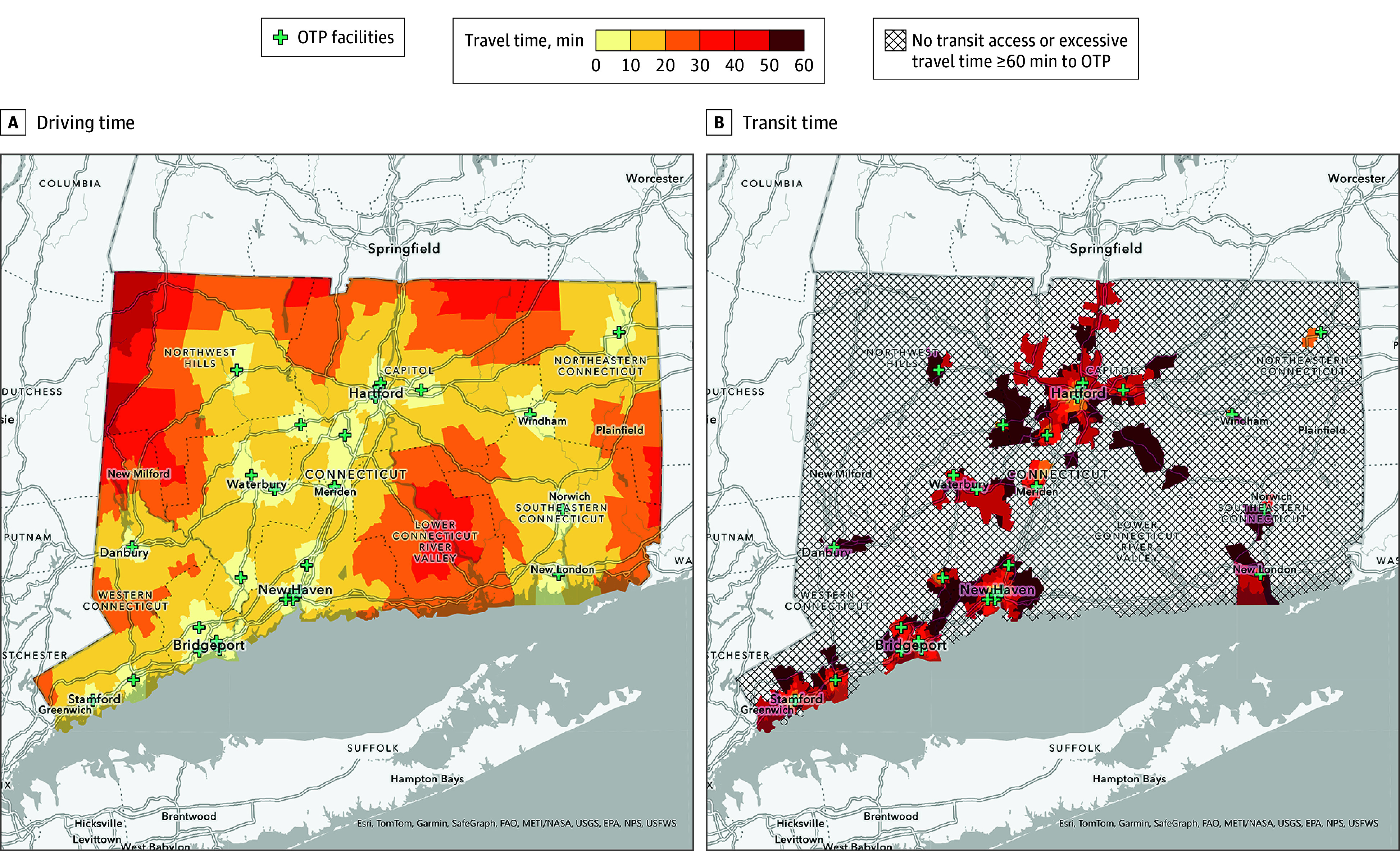
Maps of Travel Time to the Closest Opioid Treatment Program (OTP) Facilities Maps show personal vehicle travel time (A) and public transit travel time (B) in Connecticut. Text reading Esri, TomTo, Garmin, SafeGraph, FAO, METI/NASA, USGS, EPA, NPS, USFWS is an autogenerated label indicating the basemap source in ESRI ArcGIS Pro (version 3.1.3) added by default.

From the centroid of the 2702 CBGs in Connecticut, the median (IQR) travel time to the closest OTP was 11.0 (7.5-16.3) minutes by personal vehicle and 41.7 (31.0-49.5) minutes via public transit, with 1431 CBGs (53%) lacking access to transit services or requiring excessively long travel times. In CBGs where public transit was available, public transit travel time to the nearest OTP was longer than travel via personal vehicle, and the median (IQR) ratio between public transit to personal vehicle time was 5.1 (4.1-6.4). In Figure 1, we present isochrone maps visually representing the CBGs across gradations of travel time via personal vehicle and public transit.

**Figure 2.**
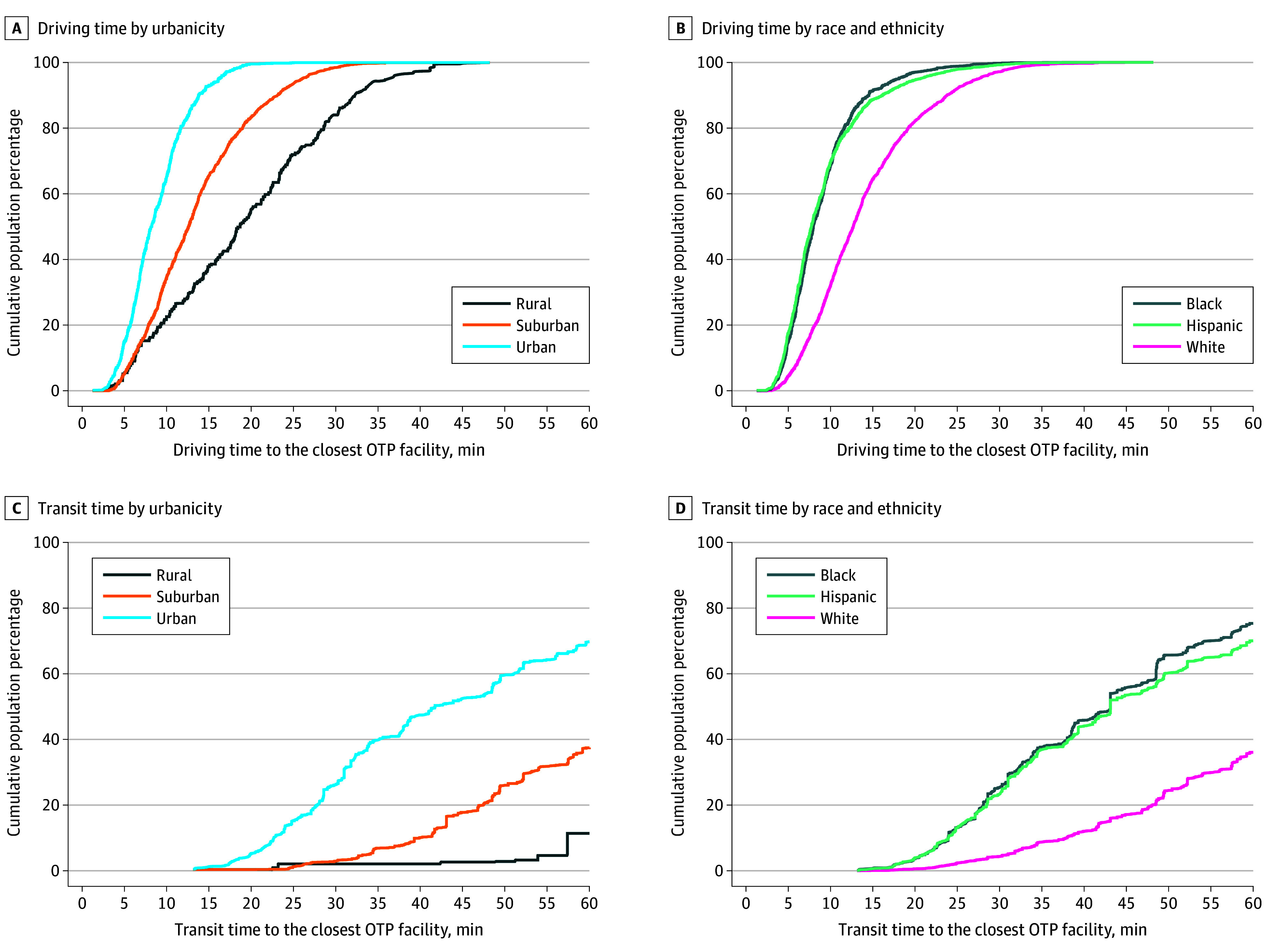
Travel Time to the Closest Opioid Treatment Program (OTP) Facility by Population Characteristics

The results of our analysis of travel time to the nearest OTP across different CBG characteristics and demographic differences are presented in Table 1. Unsurprisingly, given the predominantly urban locations of OTPs, travel times to the nearest OTP increased along the urban-rural gradient. They rose from a median (IQR) of 8.2 (6.1-11.0) minutes for personal vehicle travel and 32.3 (26.6-44.9) minutes, with 236 trips (28%) unavailable, for public transit in urban CBGs, to 19.4 (12.4-27.9) minutes for personal vehicle travel and 57.4 (52.6-57.4) minutes for public transit, with 219 trips (89%) unavailable, in rural CBGs. Rural CBGs also had a higher median (IQR) ratio of public transit to personal vehicle travel time (7.7 [6.0-9.2]) compared with suburban (5.4 [4.2-6.7]) and urban (4.8 [4.1-5.9]) CBGs. The cumulative incidence plots for residents of rural, suburban, and urban CBGs for personal vehicle travel and public transit travel are shown in Figure 2.

**Table 1.** Descriptive Statistics of Driving and Public Transit Travel Time to the Closest Opioid Treatment Program Facilities

Characteristics	Sample size, No.	Travel time, median (IQR), min		CBGs with >60-min public transit or no trip available, No. (%) (N = 2702)	Ratio of public transit to driving travel time, median (IQR)
		Driving	Public transit		
Entire study area	2702	11.0 (7.5-16.3)	41.7 (31.0-49.5)	1431 (53)	5.1 (4.1-6.4)
Urban, suburban, and rural areas					
Urban	845	8.2 (6.1-11.0)	32.3 (26.6-44.9)	236 (28)	4.8 (4.1-5.9)
Suburban	1611	12.5 (8.7-17.5)	46.8 (39.3-52.2)	976 (61)	5.4 (4.2-6.7)
Rural	246	19.4 (12.4-27.9)	57.4 (52.6-57.4)	219 (89)	7.7 (6.0-9.2)
Households without cars, %					
<5	1329	13.7 (10.1-19.1)	48.6 (41.2-52.3)	933 (70)	4.8 (3.9-5.9)
≥5	1373	8.6 (6.0-12.6)	37.8 (28.5-48.5)	498 (36)	5.2 (4.2-6.6)
Population below the poverty level, %					
<25	2381	11.7 (8.2-17)	43.9 (34.0-51.7)	1371 (58)	5.1 (4.1-6.3)
≥25 to <50	272	6.6 (4.9-9.2)	30.3 (24.0-39.3)	53 (19)	5.1 (4.1-6.6)
≥50	49	6.9 (5.2-8.6)	31.7 (26.3-43.1)	7 (14)	5.6 (4.4-6.3)
Hispanic population, %					
<25	2027	12.8 (9.2-18.1)	47.6 (37.8-52.2)	1294 (64)	5.0 (3.9-6.2)
≥25 to <50	457	7.4 (5.6-10.2)	36.2 (28.5-45.5)	114 (25)	5.3 (4.4-6.7)
≥50 to <75	187	6.1 (4.7-7.9)	31 (25.2-39.3)	21 (11)	5.5 (4.5-6.6)
≥75	31	5.5 (4.6-6.7)	25.8 (22.3-31.0)	2 (6)	4.6 (4.0-5.9)
Non-Hispanic Black population, %					
<25	2345	11.9 (8.1-17.2)	43.1 (33.0-51.5)	1385 (59)	5.2 (4.2-6.5)
≥25 to <50	264	7.1 (5.4-9.5)	33.2 (27.1-43.9)	41 (16)	5.0 (4.1-6.5)
≥50 to <75	73	7.7 (6.0-9.7)	38.1 (27.2-46.3)	3 (4)	4.4 (3.8-5.6)
≥75	20	9.7 (8.6-10.4)	38.6 (34.0-48.5)	2 (10)	4.3 (3.8-4.7)
Non-Hispanic White population, %					
<25	359	6.6 (4.9-8.5)	30.8 (24.8-39.3)	22 (6)	4.0 (2.7-4.8)
≥25 to <50	372	7.5 (5.5-10.0)	38.8 (31.0-47.6)	79 (21)	4.5 (2.2-5.7)
≥50 to <75	665	10.5 (7.7-14.4)	45.6 (34.4-52.2)	351 (53)	4.2 (2.1-5.2)
≥75	1306	14.5 (10.7-20.1)	49.3 (42.3-53.9)	979 (75)	3.8 (1.7-5.0)
No. of overdoses/100 000 persons in 2019-2021					
<50	1202	12.8 (9.2-18)	43.9 (34.5-50.4)	727 (60)	4.6 (3.8-5.8)
≥50 to <100	454	11 (7.3-16.3)	39.3 (30.6-49.4)	258 (57)	5.4 (4.1-6.6)
≥100 to <200	557	9.9 (7.0-14.6)	42.1 (31-49.5)	280 (50)	5.4 (4.4-6.5)
≥200	489	8.2 (5.9-11.7)	37.6 (27.8-48.5)	166 (34)	5.4 (4.4-6.7)

Abbreviation: CBGs, census block groups.

Moving from CBGs with higher to lower proportions of minoritized populations, median travel times via either transport modality to the nearest OTP mirrored those across the urban-to-rural spectrum. The median travel time by either transportation mode was longer in the CBGs with a larger proportion of White residents. Inversely, personal vehicle and public transit travel times were shorter in CBGs with a larger proportion of Hispanic or Black residents. The cumulative incidence plots for Black, Hispanic, and White residents for personal vehicle and public transit travel are shown in Figure 2. Travel times by both transportation modes were shorter in CBGs with a higher proportion of households with incomes below the federal poverty level. In other words, economically disadvantaged areas exhibited shorter travel times for both personal vehicle and public transit.

Finally, median (IQR) travel times in the 489 CBGs with the highest per-capita overdose rates (≥200 per 100 000 residents) were 8.2 (5.9-11.7) minutes via personal vehicle and 37.6 (27.8-48.5) minutes via public transit (166 [34%] with no trips available). Median travel times by both transportation modes were longer in CBGs with lower per-capita rates of overdose deaths. We identified 98 CBGs that had poor public transit access to an OTP, low household car ownership (≥5% of households without a vehicle), and were in the highest quartile of per-capita opioid overdose deaths.

The results of our spatial regression models are presented in Table 2. In these models, both for personal vehicle and public transit, travel times to the nearest OTP were shorter from urban and suburban CBGs compared with rural CBGs and were longer in CBGs with the greater percentage of White individuals. There were no statistically significant associations between the percentage of the population living in poverty and travel times by either personal vehicle or public transit. However, CBGs with higher per-capita opioid overdose death rates were significantly associated with shorter public transit travel times, but not with personal vehicle travel times.

**Table 2.** Associations Between Driving and Public Transit Travel Time to the Closest Opioid Treatment Program Facilities

Characteristics	Unit of measure, mean (SE)	
	Model 1 (personal vehicle travel time)	Model 2 (public transit travel time)
Percentage of population White	0.006 (0.001)^a^	0.065 (0.013)^a^
Percentage of population below poverty level	−0.003 (0.002)	−0.020 (0.019)
Opioid-related death rates	−0.031 (0.021)	−0.587 (0.209)^b^
Urban (reference: rural)	−0.847 (0.218)^a^	−5.780 (2.034)^b^
Suburban (reference: rural)	−0.859 (0.179)^b^	−3.560 (1.686)^c^
Intercept	41.489 (1.921)^a^	76.221 (2.687)^a^
Observations, No.	2702	2702
Log likelihood	−4348.139	−10 337.410
Akaike information criterion	8712.278	20 690.830

^a^ *P* < .001.

^b^ *P* < .01.

^c^ *P* < .05.

## Discussion

In this cross-sectional analysis of travel time from Connecticut CBGs to the nearest OTP we found that travel via public transit took on average 5 times longer than travel via personal vehicle. Overall, more than half (53%) of Connecticut CBGs had limited public transit access to an OTP. The differential in public transit access is unsurprising given that public transit infrastructure primarily radiates out from high-density urban locations where most OTPs are often located and becomes increasingly sparse in suburban and rural areas.^32^ While prior work has highlighted the personal drive time barriers to OTPs,^14,19,20,21,33^ which are higher in rural locations, our analysis demonstrates methadone treatment may be essentially inaccessible via public transit in many parts of Connecticut. Severe OUD is often associated with fewer financial resources and higher likelihood of reliance on public transit for daily travel. Incorporating public transit access provides a more comprehensive and accurate portrayal of the transportation barriers to OTPs. Moving beyond a simple urban-rural comparison, our analysis draws attention to transportation barriers in suburban areas, where reliance on public transit may be greater than in rural regions, yet access to OTPs via public transit remains substantially lower than in urban areas. Moreover, even within urban CBGs, the median public transit travel time to an OTP was 32.3 minutes, with 28% of block groups experiencing limited transit access—compared with a median of 8.2 minutes by personal vehicle, where OTPs are accessible to 100% of CBGs by personal vehicle.

We also found that travel burden was not evenly distributed across different demographic groups. Due to structural and historical reasons, there is substantial residential segregation and racial and ethnic minority groups predominately reside in urban centers in Connecticut. This fact, in combination with practices that have concentrated OTPs in urban neighborhoods with higher percentage of Black and Hispanic residents,^34,35^ likely explains that the travel times to OTPs by public transit from CBGs with higher percentage of Black and Hispanic residents were shorter than those with higher percentage of White residents. Even when controlling for urban or suburban or rural classification of CBGs in our multivariable analysis, shorter travel time from CBGs with higher proportions of minoritized populations persisted. Thus, our findings highlight that historical and societal pressures that influence OTP locations and housing segregation may create overlooked transportation burdens for White populations with OUD. However, overall access to MOUD must be considered in its broader context in which severe disparities exist in access particularly for Black individuals compared with their White counterparts.^36^

In addition, our findings highlight that travel time analyses, incorporating public transit travel time, relative car ownership, and prevalent geographic distribution of overdose deaths, can be used to identify gaps in the treatment infrastructure through geospatial overlay analysis. As transportation is frequently cited as a barrier to initiation and retention in methadone treatment,^14,15,16,37^ it is reasonable to site interventions to increase methadone access where they are most likely to decrease travel time for people at risk of overdose, decrease the indirect costs of treatment, increase uptake and retention in methadone treatment, and reduce overdose deaths. The CBGs in Connecticut with poor public transit access, lower car ownership, and the highest per-capita overdose death rates could be targeted for expansion of OTP services, either via the location of future OTPs, expansion of the use of fixed location medication dispensing units,^38^ or mobile methadone.^39^ Locating these interventions where the current gap in access is greatest has the potential to more efficiently target public health resources. In addition, the new Substance Abuse and Mental Health Services Administration final rule issued in 2024, which allows take-home doses of methadone, offer the potential for telehealth to extend OTPs services to those who face transportation barriers to access.^40^ For Medicaid recipients, nonemergency medical transportation, which provides transport to medical appointments, including OTPs, is an option as well, although timeliness of these services has been an issue.^41^

The interventions to increase the aforementioned inadequate methadone access are all possible under the current federal regulatory structure. Yet, the potential biggest improvements in transportation access to methadone treatment would come from changes in the statutes and regulations governing methadone treatment for OUD in the US. The Modernizing Opioid Treatment Act,^42^ which was raised in the most recent session of the US Congress, would have changed federal statute to allow for office-prescribed, pharmacy-dispensed methadone. Several studies have shown comparable treatment outcomes for patients treated in these types of models^43,44^ and can extend methadone treatment access beyond fixed-location OTPs. Our results provide additional insight into the inaccessibility of the methadone for many individuals and the potential benefit of new regulatory models that improve access.

### Limitations

There are several limitations to note. First, our analysis is at the CBG level, and may not estimate individual-level transportation access to OTPs. Second, we used per-capita overdose death as a proxy for OUD treatment need, which may not accurately represent actual prevalence of untreated OUD. Nevertheless, there are no better estimates of prevalence of OUD treatment need over small areas, and per-capita overdose death rate has been used as a proxy in similar work.^20,23,31,45^ Our analysis also does not reflect potential changes in OTP locations or per-capita opioid overdose death rates over time. Third, our analysis does not consider access to other forms of MOUD, especially buprenorphine. While buprenorphine access is important and is not tied to OTPs, it does not replace adequate access to methadone for all individuals with OUD. Methadone treatment has superior outcomes to buprenorphine and will be the preferred treatment modality for many individuals with OUD, especially among those using high-potency synthetic opioids, such as fentanyl. Fourth, our analysis treats all OTPs equally and does not account for variation in OTP quality, practice, or ability to accommodate new patients. We know that individuals with OUD will travel farther to an OTP that meets their specific treatment needs and preferences.^33^ Fifth, although our analysis assumes that individuals travel from their residential locations (ie, CBGs) to OTPs, daily mobility patterns can be more complex, and individuals may access OTPs from locations other than their homes^46,47,48^ and we did not incorporate travel time uncertainty in our analysis, which can increase perceived travel time and can be an additional driver of inequity of access.^31,49^ Similarly, we could not account for the possible access to OTPs across state lines, although this option would be limited to people accessing methadone via private health insurance or self-pay. Sixth, our analysis is limited to transportation access in one state and therefore may have limited generalizability to other states with different geographic densities of OTPs, different public transit infrastructures, and different demographics across CBGs. Conversely, Connecticut does have some geographic attributes (mix of urban, suburban, and rural CBGs with diversity of socioeconomic status and demographics), OTPs focused in urban centers, and opioid-overdose patterns, now dominated by fentanyl-involved overdoses, that will be mirrored in other states.

## Conclusions

In this cross-sectional study, we found that travel times in Connecticut via public transit to methadone treatment in 2023 were substantially longer than travel time via personal vehicle and, for many rural and suburban locations, methadone treatment was inaccessible via public transit. Current federal statutes and regulations governing methadone, which favor in-person daily treatment at fixed-location OTPs, likely contribute to these transportation barriers. We also demonstrated the potential of using advanced geospatial and transportation analyses to identify novel interventions that lower transportation barriers to OTPs and increase methadone treatment access.

## Notes

Supplement 1. eMethods. eReferences.

Supplement 2. Data Sharing Statement
