# Supplementary material for: Travel Time to Methadone Treatment Via Personal Vehicle vs Public Transit
Source: JAMA Netw Open. 2026 Feb 3;9(2):e2557361. doi: 10.1001/jamanetworkopen.2025.57361 (PMC12869344; doi:10.1001/jamanetworkopen.2025.57361)
Supplement: Supplement 1. — eMethods. eReferences. [file jamanetwopen-e2557361-s001.pdf]

Supplemental Online Content

Howell BA, Kim J, Thornhill TA, et al. Travel time to methadone treatment via personal vehicle vs public transit. *JAMA Netw Open.* 2026;9(2):e2557361.
doi:10.1001/jamanetworkopen.2025.57361

eMethods

eReferences

This supplemental material has been provided by the authors to give readers additional information about their work.

© 2026 Howell BA et al. *JAMA Network Open*.

eMethods. Spatial Regression Model Specification

This cross-sectional study examines the association between key sociodemographic variables and travel times by private vehicle (driving) and public transit. The spatial regression approach offers a straightforward yet effective method for exploring cross-sectional relationships and identifying which sociodemographic groups are significantly associated with longer driving or transit travel times.

Due to the significant spatial autocorrelation observed in the OLS residuals (Global Moran's I, p < 0.001, using a K = 5 nearest neighbors structure) and the inherently spatial nature of our unit of analysis (census block groups), combined with Lagrange Multiplier diagnostics indicating statistically significant spatial error dependence, the spatial error model was selected as the appropriate analytical framework (Anselin, 1988a; Bivand et al., 2008). The spatial error model assumes that unobserved factors associated with the dependent variable are spatially correlated across neighboring observations and that this correlation follows the neighborhood structure defined by the spatial weights matrix, such as the K-nearest neighbors (K = 5) matrix used in this study (Anselin, 1988b).

$$PVTT_i = \beta_0 + \beta_1 Wh_i + \beta_2 Bpov_i + \beta_3 Odr_i + \beta_4 Urban_i + \beta_5 Suburban_i + \varepsilon_i,$$

$$where\ \varepsilon_i = \lambda W \varepsilon_i + u_i \ldots (1)$$

$$PTTT_i = \beta_0 + \beta_1 Wh_i + \beta_2 Bpov_i + \beta_3 Odr_i + \beta_4 Urban_i + \beta_5 Suburban_i + \varepsilon_i,$$

$$where\ \varepsilon_i = \lambda W \varepsilon_i + u_i \ldots (2)$$

Equations (1) and **(2)** present the spatial error model specification. β_0 denotes the intercept, and β_1 through β_5 represent the regression coefficients for the sociodemographic variables. The parameter λ is the spatial autoregressive coefficient, and W is the spatial weights matrix that defines the neighborhood structure. In this study, we used a row-standardized K-nearest neighbors (K = 5) spatial weights matrix constructed from centroid-to-centroid Euclidean distances. The choice of K = 5 ensures that each block group has a consistent number of neighbors and avoids isolated units, particularly given that several block groups with zero population were excluded from the analysis.

The disturbance term u_i is assumed to follow an independent and identically distributed normal distribution, $u \sim N(0, \sigma^2 I)$. The composite error term ε_i represents the spatially correlated component of the residuals generated through the autoregressive structure $\varepsilon = \lambda W \varepsilon + u$, which captures unobserved influences across neighboring areas (Bivand et al., 2008).

© 2026 Howell BA et al. *JAMA Network Open*.

Variance Inflation Factor (VIF) scores from the ordinary least squares (OLS) regression models indicated no evidence of multicollinearity among the explanatory variables. A total of 2,702 census block groups were included in the regression analysis after excluding 9 block groups with zero population, which therefore lacked socioeconomic data. The model parameters were estimated using the maximum likelihood (ML) method. The full results of the two spatial regression models, including parameter estimates and diagnostics, are reported in Table 2 of the main text.

eReferences

Bivand, R. S., Pebesma, E. J., & Gomez-Rubio, V. (2008). *Applied spatial data analysis with R*. Springer New York. https://doi.org/10.1007/978-1-4614-7618-4

Anselin, L. (1988a). Lagrange multiplier test diagnostics for spatial dependence and spatial heterogeneity. *Geographical analysis*, 20(1), 1-17. https://doi.org/10.1111/j.1538-4632.1988.tb00159.x

Anselin, L. (1988b). *Spatial econometrics: methods and models*. Springer Dordrecht. https://doi.org/10.1007/978-94-015-7799-1

© 2026 Howell BA et al. *JAMA Network Open*.
