# Supplementary material for: Travel Time to Methadone Treatment Via Personal Vehicle vs Public Transit
Source: JAMA Netw Open. 2026 Feb 3;9(2):e2557361. doi: 10.1001/jamanetworkopen.2025.57361 (PMC12869344; doi:10.1001/jamanetworkopen.2025.57361)
Supplement: Supplement 2. — Data Sharing Statement [file jamanetwopen-e2557361-s002.pdf]

Data Sharing Statement

Howell. Travel Time to Methadone Treatment Via Personal Vehicle vs Public Transit. *JAMA Netw Open*. Published February 03, 2026. doi:10.1001/jamanetworkopen.2025.57361

Data
Data available: Yes
Data types: Deidentified participant data, Data dictionary
How to access data: Interested parties can email benjamin.howell@yale.edu
When available: With publication

Supporting Documents
Document types: None

Additional Information
Who can access the data: researchers whose proposed use of the data has been approved
Types of analyses: for any purpose
Mechanisms of data availability: after approval of a proposal and with a signed data access agreement
